# Assessment of a combined strategy of seasonal malaria chemoprevention and supplementation with vitamin A, zinc and Plumpy’Doz™ to prevent malaria and malnutrition in children under 5 years old in Burkina Faso: a randomized open-label trial (SMC-NUT)

**DOI:** 10.1186/s13063-021-05320-7

**Published:** 2021-05-24

**Authors:** Paul Sondo, Marc Christian Tahita, Toussaint Rouamba, Karim Derra, Bérenger Kaboré, Cheick Saïd Compaoré, Florence Ouédraogo, Eli Rouamba, Hamidou Ilboudo, Estelle Aïssa Bambara, Macaire Nana, Edmond Yabré Sawadogo, Hermann Sorgho, Athanase Mwinessobaonfou Somé, Innocent Valéa, Prabin Dahal, Maminata Traoré/Coulibaly, Halidou Tinto

**Affiliations:** 1 Institut de Recherche en Sciences de la Santé (IRSS)/Clinical Research Unit of Nanoro (CRUN), Nanoro, Burkina Faso; 2 National Malaria Control Program, Ministry of Health, Ouagadougou, Burkina Faso; 3 Nutrition Direction, Ministry of Health, Ouagadougou, Burkina Faso; 4 Health District of Nanoro, Ministry of Health, Nanoro, Burkina Faso; 5 Infectious Diseases Data Observatory (IDDO), University of Oxford, Oxford, UK

**Keywords:** Malaria, Malnutrition, Seasonal chemoprevention, Plumpy’Doz™, Vitamin A, Zinc, Randomized controlled trial

## Abstract

**Background:**

Malaria and malnutrition represent major public health concerns worldwide especially in Sub-Sahara Africa. Despite implementation of seasonal malaria chemoprophylaxis (SMC), an intervention aimed at reducing malaria incidence among children aged 3–59 months, the burden of malaria and associated mortality among children below age 5 years remains high in Burkina Faso. Malnutrition, in particular micronutrient deficiency, appears to be one of the potential factors that can negatively affect the effectiveness of SMC. Treating micronutrient deficiencies is known to reduce the incidence of malaria in highly prevalent malaria zone such as rural settings. Therefore, we hypothesized that a combined strategy of SMC together with a daily oral nutrients supplement will enhance the immune response and decrease the incidence of malaria and malnutrition among children under SMC coverage.

**Methods:**

Children (6–59 months) under SMC coverage receiving vitamin A supplementation will be randomly assigned to one of the three study arms (a) SMC + vitamin A alone, (b) SMC + vitamin A + zinc, or (c) SMC + vitamin A + Plumpy’Doz™ using 1:1:1 allocation ratio. After each SMC monthly distribution, children will be visited at home to confirm drug administration and followed-up for 1 year. Anthropometric indicators will be recorded at each visit and blood samples will be collected for microscopy slides, haemoglobin measurement, and spotted onto filter paper for further PCR analyses. The primary outcome measure is the incidence of malaria in each arm. Secondary outcome measures will include mid-upper arm circumference and weight gain from baseline measurements, coverage and compliance to SMC, occurrence of adverse events (AEs), and prevalence of molecular markers of antimalarial resistance comprising *Pfcrt*, *Pfmdr1*, *Pfdhfr,* and *Pfdhps*.

**Discussion:**

This study will demonstrate an integrated strategy of malaria and malnutrition programmes in order to mutualize resources for best impact. By relying on existing strategies, the policy implementation of this joint intervention will be scalable at country and regional levels.

**Trial registration:**

ClinicalTrials.gov NCT04238845. Registered on 23 January 2020

https://clinicaltrials.gov/ct2/show/NCT04238845

## Administrative information

The order of the items has been modified to group similar items (see http://www.equator-network.org/reporting-guidelines/spirit-2727-statement-defining-standard-protocol-items-for-clinical-trials/).

**Table:** 

Title {1}	**Assessment of a combined strategy of seasonal malaria chemoprevention and supplementation with Vitamin A, Zinc and Plumpy’Doz™ to prevent malaria and malnutrition in children under five years old in Burkina Faso: a randomized open label trial (SMC-NUT)**
Trial registration {2a and 2b}.	NCT04238845 23/01/2020 Clinicaltrials.gov
Protocol version {3}	01/05/2020, Version 02
Funding {4}	European & Developing Countries Clinical Trials Partnership (EDCTP) through the EDCTP career Development Fellowship TMA2018CDF-2365
Author details {5a}	^1^Institut de Recherche en Sciences de la Santé (IRSS)/Clinical Research Unit of Nanoro (CRUN) ^2^National Malaria Control Program, Ministry of health, Burkina Faso ^3^Nutrition Direction, Ministry of Health Burkina Faso ^4^ Health District of Nanoro, Ministry of Health of Burkina Faso ^5^Infectious Diseases Data Observatory (IDDO), University of Oxford, Oxford, United Kingdom
Name and contact information for the trial sponsor {5b}	Institut de Recherche en Sciences de la Santé (IRSS) 03BP7192 Ouagadougou 03 Tel : +22625363215 / +22625363364 Fax : +22625360394
Role of sponsor {5c}	The sponsor is responsible for the study protocol development and ensures that proper arrangements are in place to initiate, manage and report on the study.

## Introduction

### Background and rationale {6a}

Malaria is hyper-endemic in Burkina Faso and many sub-Saharan African countries [1]. According to the National Malaria Control Program (NMCP) approximately 11,915,816 malaria cases and 4144 malaria-related deaths were reported in 2017 in Burkina Faso [2, 3]. Numerous malaria control interventions have been implemented in Burkina Faso in order to achieve the 2020 Sustainable Development Goal (SDG) 3 [4]. Seasonal malaria chemoprevention (SMC) ranks among the largest and reliable interventions for malaria control in countries with marked seasonality of malaria transmission. The goal of this intervention is to treat any existing infections and maintain protective drug concentrations in the blood throughout the complete high transmission season. This preventive measure involves administering antimalarial drugs (amodiaquine-sulfadoxine-pyrimethamine) to children aged 3–59 months on a monthly basis during the high transmission peak. SMC is recommended by the World Health Organization (WHO) and is known to reduce malaria morbidity by 30 to 83% [5–8]. In Burkina Faso, SMC is implemented from July/August to October/November each year. Despite the implementation of this strategy, the burden of malaria and associated mortality is still very high in this target population in Burkina Faso. This raises questions about other hidden factors that can negatively affect the effectiveness of SMC intervention.

At the same time, malnutrition is a major cause of childhood morbidity and mortality in low- and middle-income countries such as Burkina Faso [9, 10]. Malnutrition can lower immune system leading to increased susceptibility to malaria in the population. The Nutrition Direction, which is the agency established to address malnutrition problems in Burkina Faso, currently aims to scale-up Community Management of Acute Malnutrition (CMAM) [11, 12]. This approach involves timely detection of severe acute malnutrition in the community and provision of treatment for those without medical complications with ready-to-use therapeutic foods or other nutrient-dense foods at home. Both malaria and malnutrition are highly prevalent in rural settings. Furthermore, malaria seasonal peak coincides with period of food crisis in the country (as it is the farming period) suggesting that malnutrition may influence malaria incidence during that period. Children under 5 years again represent the most vulnerable group. Importantly, treating micronutrient deficiencies is known to reduce malaria morbidity and mortality [13, 14]. Plumpy’Doz™ is specifically formulated for the prevention of malnutrition in children from 6 months of age and adults [15, 16]. Evidence also shows that vitamin A-zinc supplementation reduce malaria morbidity [13]. A reduction by 20–30% of malaria incidence through micronutrient supplementation such as vitamin A-zinc has also been reported [13, 14].

These aspects underlie the idea of the proposed SMC-NUT project. This combined strategy could reduce the burden of malnutrition, enhance the immune system, and therefore decrease the susceptibility and severity of malaria in children under SMC coverage. This project will serve as pilot study for integrated strategy in order to mutualize resources for better impact. By relying on existing strategies, the policy implementation of this joint intervention will be scalable at country and regional levels.

### Objectives {7}

The overarching aim of the study is to study the impact of nutritional supplementation on different malaria indicators. The primary objectives include (i) assessing in a randomized open-label trial whether SMC + vitamin A + zinc or SMC + vitamin A + Plumpy’Doz™ is more effective and safe in reducing uncomplicated malaria incidence, severe malaria incidence, and related mortality compared to the SMC + vitamin A alone and (ii) to investigate the impact of the combined strategy in reducing the burden of malnutrition through reduction of anaemia incidence, mid-upper arm circumference, and weight gain.

Secondary objectives include (i) to assess the impact of each treatment arm on the circulating parasite population through monitoring the temporal trend of antimalarial resistance molecular markers *pfcrt*, *pfmdr1*, *dhfr,* and *dhps*; (ii) to assess the coverage and compliance to SMC in the study area; and (iii) to quantify the overall occurrence of adverse events (AEs).

### Trial design {8}

This will be a randomized open-label trial. Children (6–59 months) under SMC coverage receiving vitamin A supplementation will be randomly assigned to one of the three study arms (a) SMC + vitamin A alone, (b) SMC + vitamin A + zinc, or (c) SMC + vitamin A + Plumpy’Doz™ using 1:1:1 allocation ratio.

## Methods: Participants, interventions, and outcomes

### Study setting {9}

The study will take place in Nanoro, a regional direction of the Institut de Recherche en Sciences de la Santé (IRSS) in Burkina Faso. Nanoro is a rural area situated in the central part of Burkina Faso, around 85 km from Ouagadougou, the capital city of Burkina Faso. Data will be collected in four villages (Soaw, Rakalo, Zoetgomdé, and Kalwaka) covered by two peripheral health facilities (Soaw and Zoetgomdé). Malaria is hyper-endemic in Nanoro with a marked seasonality of transmission making it an ideal place for studying the impact of SMC intervention [17, 18]. Through its Health and Demographic Surveillance System (HDSS) platform, Nanoro offers an ideal opportunity to conduct such a study requiring a long-term follow-up period [19].

### Eligibility criteria {10}

Children will be included in the study if they fully comply with the following criteria: (i) children under both interventions coverage (SMC, vitamin A supplementation), i.e. 6–59 months old; (ii) resident within HDSS coverage area; (iii) ability to complete the study follow-up period; and (iv) written consent obtained from parents. Exclusion criteria are (i) individual not under both interventions coverage, (ii) children under both interventions coverage who did not receive any of the treatment (amodiaquine-sulfadoxine-pyrimethamine or vitamin A), (iii) ill individual at the time of the enrolment including uncomplicated/severe malaria or severe malnutrition, (iv) known allergy to vitamin A or zinc or Plumpy’Doz™, (v) planned travel or inability to complete the study follow-up, and (vi) unwillingness to participate to the study.

### Who will take informed consent? {26a}

Before starting the study, the community will be informed on the purpose of the study through the community leaders and a general consent will be obtained. A written informed consent will be obtained from all the study participants before enrolment. Parent/guardians of eligible children will receive in advance a copy of the study information sheet and the consent form from community health workers which they are welcome to discuss with their family members and friends. The information sheet will describe relevant information related to the study such as the purpose of the study, the study procedures, the confidentiality of recorded data, and the risks and benefits of the participation.

As the study involves minors (children aged 6–59 months), the parents or guardians will be asked to sign the consent to participate in the study.

If a parent or guardian is unable to read or write, a signature from an impartial adult witness present during the informed consent discussion and a thumb print of the parent/guardian will be obtained. The impartial witness will ensure that the reading of the consent form to the potential participant has been performed accurately, and the parent/guardian has had the opportunity to ask questions and therefore confirm that the parent/guardian has given consent freely.

### Additional consent provisions for collection and use of participant data and biological specimens {26b}

In the inform consent procedure, parent/guardians will be asked about future investigations on the unused blood samples. They will be explained that future investigations will only focus on the understanding of the parasite resistance mechanisms. No genetic testing about human host will be performed.

### Interventions

#### Explanation for the choice of comparators {6b}

Seasonal malaria chemoprevention (SMC) is one of the routine malaria control intervention and all children from 3 to 59 months old in the country are expected to be covered. In addition, vitamin A supplementation campaign is part of the national strategy for prevention of malnutrition in children (6–59 months) in Burkina Faso. The comparator represents children receiving both interventions as part of routine strategy for preventing malaria and malnutrition in Burkina Faso.

#### Intervention description {11a}

**Fig. 1:**
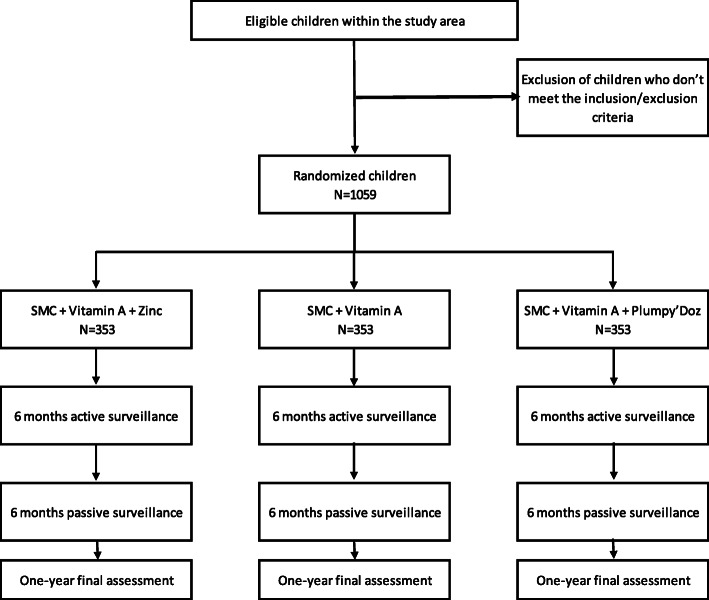
Study flowchart diagram

All interventions will be implemented in strict compliance with preventive measures against the spread of COVID-19 disease such as wearing masks and the use of hydroalcoholic gel. Prior to SMC first round, the targeted population living across HDSS area will be identified from the dataset of the last update round (sampling frame). Potential study participants will be randomly drawn from this sampling frame (Fig. 1).

Home visit will be performed to confirm the presence of the child, notify, and inform parents of eligible participants about the study and where possible a written informed consent will be obtained. Absent children or children whose parents are not willing to participate will not be considered and will be replaced by other eligible children from the list.

##### SMC intervention

The SMC intervention is based on a complete dose administration of amodiaquine-sulfadoxine-pyrimethamine and will be implemented nationally by the NMCP. According to the national malaria cases management guidelines, amodiaquine-sulfadoxine-pyrimethamine (AQSP) will be administered once daily over 3 days [2]. The first dose will be administered under observation by the community health workers (CHW), while the remaining doses will be taken unobserved during the following 2 days.

##### Vitamin A supplementation

Similarly, vitamin A supplementation will be organized by the Nutrition Direction. Vitamin A will be administered as single dose of 200,000 UI per child under supervision by the CHW. All these interventions (SMC and vitamin A supplementation) will be implemented through the operational entity of the Ministry of Health in the study area which is the health district of Nanoro.

##### Plumpy’Doz™ supplementation

Plumpy’Doz™ is a ready-to-use nutritional lipid-based nutrient supplement that can be used at home without prior preparation. However, parents/guardians will be advised about the mode of administration of the product, with special interest paid to hygiene precautions. Each child will receive 1 sachet/day for 4 months (SMC period).

##### Zinc supplementation

Zinc supplementation (the equivalent of 10 mg of elemental zinc) will be provided on a daily basis during 6 days per week for 4 months (SMC period).

#### Criteria for discontinuing or modifying allocated interventions {11b}

It is anticipated that all of the product will be well tolerated. However, an efficient pharmacovigilance system will be put in place to ensure prompt reporting and management of adverse events requiring medical care and supplementation will be interrupted in case of frequent occurrence of related serious adverse events.

#### Strategies to improve adherence to interventions {11c}

All the products that will be used in this study are already used as part of routine management and prevention of malnutrition. Before enrolment and at each visit, we will insist on communication/sensitization about special care requirements for investigational product.

#### Relevant concomitant care permitted or prohibited during the trial {11d}

No concomitant care and routine interventions are prohibited during the trial. However, all concomitant care and interventions during the trials will be recorded.

#### Provisions for post-trial care {30}

There is no anticipated harm and compensation for trial participation and no anticipated provision for post-trial care.

#### Outcomes {12}

Primary outcomes include (i) the relative incidence of clinical malaria, severe malaria, and anaemia in SMC + vitamin A group (control group) compared to each of the two intervention arms, i.e. SMC + vitamin A + supplement and (ii) the mid-upper arm circumference gain and weight gain from the enrolment to the end of the 12-month study period in the intervention groups compared to the control group. Secondary outcomes include (i) the prevalence of mutant alleles of *pfcrt, pfmdr1, pfdhfr*, and *pfdhps* from the enrolment to the end of the 6-month study period in malaria-positive sample and (ii) SMC coverage in the area which will be determined as the ratio of children who actually received the intervention to the predetermined sample of eligible children from the HDSS dataset. (iii) Compliance will be determined by calculating, for each participant, the ratio between the number of tablets/packets taken and the number of tablets/packets that the patient should have taken. Two groups of participants will be described: compliant = 100% treatment compliance and non-compliant = other cases, and (iv) the incidence of adverse events in each study arms.

#### Participant timeline {13}

Children will be included after confirmation of the administration of vitamin A and the first dose of AQSP of the first SMC round. Eligible children will be followed-up for 1 year (Fig. 1). This will consist of an active surveillance system during the first 6 months to fully cover the malaria peak followed by a passive surveillance system for the rest of the period. During the active surveillance period, scheduled visits will be performed monthly. Anthropometric indicators and occurrence of adverse events will be recorded at each visit. Blood samples will be collected for microscopy slides, haemoglobin measurement, and spotted onto filter paper for further polymerase chain reaction (PCR) analyses.

**Table:** 

	Study period									
	Enrolment	Allocation	Post-allocation							
Timepoint**	− t_**1**_	0	M_**1**_	M2	M3	M4	M5	M6	M7–M11	M12 (Close-out)
**Enrolment**										
**Eligibility screen**	X									
**Informed consent**	X									
**Confirmation of SMC drug intake**		X								
**Confirmation of vitamin A intake**		X								
**Allocation**		X								
**Interventions**										
**[SMC + vitamin A + Plumpy’Doz]**			X	X	X	X				
**[SMC + vitamin A + zinc]**			X	X	X	X				
**[SMC + vitamin A]**			X	X	X	X				
**Assessments**										
**[Body temperature]**			X	X	X	X				X
**[Weight]**			X	X	X	X	X.	X		X
**[Height]**			X	X	X	X	X	X		X
**Malaria RDT**			X	X	X	X	X	X		X
**Malaria microscopy**			X	X	X	X	X	X		X
**MUAC**			X	X	X	X	X	X		X
**Haemoglobin measurement**			X	X	X	X	X	X		X
**Blood spots for PCR**			X	X	X	X	X	X		X
**Adverse events**			X	X	X	X	X	X		X

Axillary temperature will be determined using an electronic clinical thermometer. Mid-upper arm circumference (MUAC) will be measured at the mid-point between the tip of the shoulder and the tip of the elbow. Height will be measured using an appropriate Rolla-meter and recorded in centimetres. Weight will be measured using a baby scale for children aged 6 months to 2 years old, and a children’s sitting/standing scale for children over 2 years old. Blood will be collected by finger prick for haemoglobin (Hb) measurement, malaria Rapid Diagnostic Test (RDT), microscopy, and on filter paper for later PCR analyses. A HemoCue® 201^+^ will be used for photometric measurement of Hb in the field following the manufacturer’s procedure. Thick and thin blood films will be stained with Giemsa 3% for 30 min and examined with light microscope. A slide will be declared negative if the examination of 100 thick-film fields does not reveal the presence of asexual parasites. PCR analyses concern a genotyping of amodiaquine resistance molecular markers (*pfcrt* and *pfmdr1*), sulfadoxine resistance marker (*pfdhps*), and pyrimethamine resistance marker (*pfdhfr*) on malaria-positive samples [20]. Participant timeline can be summarized as follows:

#### Sample size {14}

Sample size calculation was carried out with incidence of malaria as the primary outcome measure under the assumption that the expected proportion of children 6–59 months who will acquire malaria in the absence of seasonal malaria chemoprevention (SMC) during the rainy season in Burkina Faso is 50%. The introduction of SMC is expected to reduce this proportion to 30%. The SMC programme together with nutritional intervention is expected to reduce this to 20%. The aim is to demonstrate that SMC + nutritional supplement arm is superior to the SMC alone arm in reducing the incidence of malaria. On this basis, a one-side α of 0.025 was chosen. Type II error was controlled at 20% (i.e. β = 0.20 or 80% power). Based on these parameters, a sample size of 294 was required for each pair-wise comparison (SMC + vitamin A vs SMC + vitamin A + zinc, and SMC + vitamin A vs SMC + vitamin A + Plumpy’Doz™). Hence, an overall sample size for the study was estimated at 882 (3 × 294) to detect a 10% difference in risk of malaria occurrence between SMC alone group and any of SMC + supplement group. Further assuming that 20% of the individuals will be lost to follow-up, an adjusted sample size of 353 children per arm was required. The final estimated sample size for this study is thus 1059 children.

#### Recruitment {15}

The study will rely on existing Health and Demographic Surveillance System (HDSS) for identification of eligible participants. All the participants will be recruited within the coverage area of the Nanoro HDSS.

#### Assignment of interventions: allocation

##### Sequence generation {16a}

This will be a randomized open-label trial. A computer-generated randomization list will be pre-defined. Only the central pharmacist will have access to the randomization list.

##### Concealment mechanism {16b}

Each random number and corresponding treatment will be sealed in an opaque envelope which will only be opened by CHW after enrolment of the participant.

##### Implementation {16c}

The central pharmacist of IRSS-DRCO will generate the allocation sequence. Participants will be enrolled by community health workers who will also assign participants to interventions after confirmation of all eligibility criteria.

#### Assignment of interventions: Blinding

##### Who will be blinded {17a}

Not applicable as the study is an open-label trial.

##### Procedure for unblinding if needed {17b}

Not applicable as the study is an open-label trial.

### Data collection and management

#### Plans for assessment and collection of outcomes {18a}

Data collection and measurement of study parameters will be performed according the developed standard operating procedures. Axillary temperature will be determined using an electronic clinical thermometer. Mid-upper arm circumference (MUAC) will be measured at the mid-point between the tip of the shoulder and the tip of the elbow. Height will be measured using an appropriate Rolla-meter and recorded in centimetres. Weight will be measured using a baby scale for children aged 6 months to 2 years old, and a children’s sitting/standing scale for children over 2 years old. Blood will be collected by finger prick for haemoglobin (Hb) measurement, malaria rapid diagnostic test (RDT), microscopy, and on filter paper for later PCR analyses. A HemoCue® 201^+^ will be used for photometric measurement of Hb in the field following the manufacturer’s procedure. Thick and thin blood films will be stained with Giemsa 3% for 30 min and examined with light microscope. A slide will be declared negative if the examination of 100 thick-film fields does not reveal the presence of asexual parasites. PCR analyses concern a genotyping of amodiaquine resistance molecular markers (*pfcrt* and *pfmdr1*), sulfadoxine resistance marker (*pfdhps*), and pyrimethamine resistance marker (*pfdhfr*) on malaria-positive samples following previously published protocols.

#### Plans to promote participant retention and complete follow-up {18b}

For studies with such a long follow-up period (12 months), loss to follow-up is an important issue. Therefore, by relying on Nanoro health and demographic surveillance system (HDSS), this risk is highly minimized. In addition, 20% corrective measure was allocated to this risk in the sample size calculation to prevent it from affecting the study validity. The supplements that will be used in this study (Plumpy’Doz and zinc) are well known by the population in the management of malnutrition. However, this study will insist on communication with the community/parent/guardians before enrolment in order to improve knowledge and awareness of study product as well as the purpose of the study.

#### Data management {19}

Appropriate individual case record forms (CRFs) will be developed for each participant. Data will be double entered by two independent data clerks in a REDCAP database. Adequate procedures will be put in place to ensure accuracy, reliability, and consistency of records. Control systems will be set-up to independently record the date and time of operator entries and actions that create, modify, or delete records. All the data will be backed up and archived daily.

#### Confidentiality {27}

The medical records of the participants and information collected during this study that display their names or address details will be kept confidential at the IRSS/CRUN. This identifying information will be seen by the ethics committee for health research, or the regulatory authority (both in Burkina Faso) who gives us permission to conduct the study and make sure the study is run properly.

#### Plans for collection, laboratory evaluation, and storage of biological specimens for genetic or molecular analysis in this trial/future use {33}

Dried blood spots will be put into plastic bags and stored at room temperature. Future investigations will only focus on the understanding of the parasite resistance mechanisms. No genetic testing about human host will be performed.

### Statistical methods

#### Statistical methods for primary and secondary outcomes {20a}

Data analysis will be performed using R software package [21] or STATA 14.1 software. A statistical analysis plan will be developed within 6 months of the study commencement. In case of protocol amendment or any unexpected events affecting this plan, it may be revised during the course of the study accordingly. A final statistical analysis plan will be produced before the database lock.

#### Interim analyses {21b}

Not planned yet

#### Methods for additional analyses (e.g. subgroup analyses) {20b}

Not planned yet

#### Methods in analysis to handle protocol non-adherence and any statistical methods to handle missing data {20c}

Analysis population relating to protocol non-adherence and statistical methods to handle missing data will be defined in the statistical analysis plan that will be developed prior to database lock.

#### Plans to give access to the full protocol, participant level-data, and statistical code {31c}

Prior to the start of the study, the protocol will be registered in an international registry and published in an open access journal. The datasets analysed during the current study will be available from the corresponding author on reasonable request.

### Oversight and monitoring

#### Composition of the coordinating centre and trial steering committee {5d}

Prior to the study start, a trial steering committee will be set. This committee will include five independent members: a paediatrician, a nutritionist, a member of the Nanoro health district executive team, and representatives of the national malaria and nutrition programmes (one from each programme). The committee will monitor the evolution of the trial through regular teleconferences and physical meetings (twice during the course of the intervention) and advice on the scientific credibility and on specific issues that may arise during the course of the study. All decisions taken by this committee will be made by majority vote and will be documented.

#### Composition of the data monitoring committee, its role, and reporting structure {21a}

IRSS will contract a freelance monitor to ensure that the study is adequately monitored. The monitor will verify the best conduct of the study through phone calls with the principal investigator and other study staff. Two monitoring visits are planned: an initiation visit before the enrolment of the first participant and a close-out visit after the last patient has completed the follow-up.

#### Adverse event reporting and harms {22}

All the medical products that will be administered in this study are already used as part of either routine management or prevention of malnutrition in Burkina Faso. Therefore, it is anticipated that the study drugs will be well tolerated. However, an efficient pharmacovigilance system will be put in place to ensure prompt reporting and management of adverse events requiring medical care.

#### Frequency and plans for auditing trial conduct {23}

The study protocol is reviewed by technical committee for the examination of authorization for clinical trial requests of the national agency for pharmaceutical regulation which is the regulatory authority in Burkina Faso. An approval was obtained and the study could be subject to audit by this regulatory authority of Burkina Faso anytime if necessary.

#### Plans for communicating important protocol amendments to relevant parties (e.g. trial participants, ethical committees) {25}

Any protocol amendment will be reported to the ethical committee for health research of Burkina Faso and to the national agency for pharmaceutical regulation in Burkina Faso.

#### Dissemination plans {31a}

Dissemination of the study findings is planned not only through scientific papers and conference attendance, but also through meetings with various audiences of interest, including key stakeholders such as decision-makers and public health authorities, health workers, and local community.

## Discussion

Despite the implementation of multiple control strategies, the burden of malaria suggests the need of innovative measures either to improve the efficacy of exiting strategies or to complement them. This study will boost the efficacy of SMC intervention, one the largest and reliable malaria control interventions in Sahelian countries. Most importantly, the study will aim to generate evidence on the efficacy of nutritional supplementation to reduce the burden of malnutrition which constitutes a huge threat in sub-Sahara Africa. Thus, this study will provide critical information for policy-makers and will contribute towards the achievement of SDG 3.

The study will promote economic development and welfare in Burkina Faso. The outcomes of this project will indirectly contribute to the economic development by reducing the burden of malaria and malnutrition, which constitute significant threats to economic development in the country. As well as the wider economic threats, these issues have huge impact at the individual and community levels through reduced attendance in education, reduced working days lost due to disease, reduced household and community incomes, and increased burden on patients because of treatment costs. The combined intervention will also contribute to saving resources for the national malaria and nutrition programmes. This includes time, personnel, logistics allocated for the implementation of SMC, and nutrition prevention strategies.

This study will serve as a pilot of integrated strategies in order to federate resources for better impact. The proposed project will greatly contribute to guide national policy of two distinct entities of the Ministry of Health in their decision-making by providing evidence of the impact of an easily scalable combined intervention, i.e. the National Malaria Control Program and the National Nutrition Direction.

Finally, by relying on existing strategies, the policy implementation of this joint intervention demonstrates its public health sustainability and will be scalable at country and regional levels especially in other Sahel countries sharing similar malaria and malnutrition profile.

### Trial status

This is version 02, 01/05/2020 of the protocol. Ethical and regulatory authority approvals are obtained. Study staff are recruited and trained. At the time of submission, the recruitment of study participants began in July 13, 2020 and was expected to be completed 2 weeks later, i.e. in August 2020. Participants will be followed-up until June 2021. The trial is ongoing. The results of the study are expected in March 2022.

## Abbreviations

AQSP: Amodiaquine-sulfadoxine-pyrimethamine
CHW: Community health worker
CRF: Case record form
EDCTP: European and Developing Countries Clinical Trials Partnership
Hb: Haemoglobin
HDSS: Health and Demographic Surveillance System
IRSS: Institut de Recherche en Sciences de la Santé
NMCP: National Malaria Control Program
PCR: Polymerase chain reaction
*Pfcrt*: *Plasmodium falciparum* chloroquine resistance transporter gene
*Pfdhfr*: *Plasmodium falciparum* dihydrofolate reductase
*Pfdhps*: *Plasmodium falciparum* dihydropteroate synthase
*Pfmdr1*: *Plasmodium falciparum* multidrug resistance 1
SDG: Sustainable Development Goals
SMC: Seasonal malaria chemoprevention
WHO: World Health Organization
